# 

**Published:** 1859-08

**Authors:** 


### THIRTEENTH VOLUME

OF THE

### DENTAL REGISTER OF THE WEST,

EDITED BY

### J. TAFT and GEO. WATT.

**Issued Monthly. - - - $3 a year in Advance.**

The Volume commences in July. CONTRIBUTIONS to the pages of the Register respectfully solicited.

Exchanges and Correspondents will please address "Dental Register," or J. TAFT, Cincinnati, O; Business communications to JNO. T. TOLAND.

The Register being issued Monthly is always fresh, and in advance of all others, therefore indispensable to the practitioner who desires to keep posted up with the new inventions and improvements which are daily developed.

Neither expense nor effort will be spared to give value and variety to its contents. Articles will be illustrated with well executed engravings, whenever they will add to the interest or assist in explanation.

Its extensive circulation and frequency of issue makes it the BEST DENTAL ADVERTISING MEDIUM in the United States.

### TERMS OF ADVERTISING.

| | One Year. | 6 Mo. | 3 Mo. | 1 Insertion. |
|---|---|---|---|---|
| One Page, | $50 | $30 | $20 | $15 |
| Half | 30 | 20 | 15 | 10 |
| Quarter | 20 | 15 | 10 | 7 |
| One-Eighth | 15 | 10 | 7 | 5 |

**JNO. T. TOLAND, Pub. and Proprietor,**

38 *West Fourth Street, Cincinnati, Ohio.*

